# High-Temperature Short-Time Pasteurization System for Donor Milk in a Human Milk Bank Setting

**DOI:** 10.3389/fmicb.2018.00926

**Published:** 2018-05-11

**Authors:** Diana Escuder-Vieco, Irene Espinosa-Martos, Juan M. Rodríguez, Nieves Corzo, Antonia Montilla, Pablo Siegfried, Carmen R. Pallás-Alonso, Leónides Fernández

**Affiliations:** ^1^Banco Regional de Leche Materna, Hospital Universitario 12 de Octubre, Instituto de Investigación i+12, Madrid, Spain; ^2^Probisearch S.L., Tres Cantos, Spain; ^3^Departamento de Nutrición, Bromatología y Tecnología de los Alimentos, Universidad Complutense de Madrid, Madrid, Spain; ^4^Departamento de Bioactividad y Análisis de Alimentos, Instituto de Investigación en Ciencias de la Alimentación, CIAL (CSIC-UAM), Madrid, Spain; ^5^Sive Fluid Systems S.L., Alcalá de Henares, Spain; ^6^Servicio de Neonatología, Hospital Universitario 12 de Octubre, Instituto de Investigación i+12, Universidad Complutense de Madrid, Madrid, Spain

**Keywords:** donor milk, HTST pasteurization, microbiological quality, enzyme indicators

## Abstract

Donor milk is the best alternative for the feeding of preterm newborns when mother's own milk is unavailable. For safety reasons, it is usually pasteurized by the Holder method (62.5°C for 30 min). Holder pasteurization results in a microbiological safe product but impairs the activity of many biologically active compounds such as immunoglobulins, enzymes, cytokines, growth factors, hormones or oxidative stress markers. High-temperature short-time (HTST) pasteurization has been proposed as an alternative for a better preservation of some of the biological components of human milk although, at present, there is no equipment available to perform this treatment under the current conditions of a human milk bank. In this work, the specific needs of a human milk bank setting were considered to design an HTST equipment for the continuous and adaptable (time-temperature combination) processing of donor milk. Microbiological quality, activity of indicator enzymes and indices for thermal damage of milk were evaluated before and after HTST treatment of 14 batches of donor milk using different temperature and time combinations and compared to the results obtained after Holder pasteurization. The HTST system has accurate and simple operation, allows the pasteurization of variable amounts of donor milk and reduces processing time and labor force. HTST processing at 72°C for, at least, 10 s efficiently destroyed all vegetative forms of microorganisms present initially in raw donor milk although sporulated *Bacillus* sp. survived this treatment. Alkaline phosphatase was completely destroyed after HTST processing at 72 and 75°C, but γ-glutamil transpeptidase showed higher thermoresistance. Furosine concentrations in HTST-treated donor milk were lower than after Holder pasteurization and lactulose content for HTST-treated donor milk was below the detection limit of analytical method (10 mg/L). In conclusion, processing of donor milk at 72°C for at least 10 s in this HTST system allows to achieve the microbiological safety objectives established in the milk bank while having a lower impact regarding the heat damage of the milk.

## Introduction

Breast milk provides all the nutrients, vitamins, and minerals, required for infant growth. In addition, its content in specific bioactive compounds such as immunoglobulins, enzymes, cytokines, growth factors or hormones, also protects against infections and modulates maturation of the digestive and immune systems and neurodevelopment. Furthermore, human milk composition is highly variable (within feeds, diurnally, over lactation) in order to promote both survival and healthy development of the infant (Ballard and Morrow, 2013). Therefore, mother's own milk (MOM) is the gold standard for the feeding and nutrition of term and preterm newborn during the first months of life (Section on breastfeeding American Academy of Paediatrics, 2012).

When MOM is unavailable or is in short supply to meet the nutritional requirements of the preterm infants, which is relatively frequent in Neonatal Intensive Care Units (NICUs), the next best alternative is the use of donor human milk (DHM) (ESPGHAN Committee on Nutrition et al., 2013; DiLauro et al., 2016). The use of DHM is associated to a reduction in the incidence of necrotizing enterocolitis, protection against late-onset sepsis and improved feeding tolerance compared to formula milk in this high-risk group of infants (Quigley and McGuire, 2014; Sisk et al., 2017). Human Milk Banks (HMBs) are established to recruit and screen milk donors, collect, treat, store, and distribute donated milk. In order to provide safe DHM, implementation of very stringent quality control and quality assurance systems are required in HMBs (DeMarchis et al., 2017).

The microbiological quality of DHM is one of the main concerns in most HMBs as it will be administered mainly to preterm or sick infants, who are especially susceptible to infections. Human milk contains a site-specific microbiota, mainly composed of staphylococci, streptococci and corynebacteria, which plays an important role in the initiation and development of the neonatal gut microbiota and contribute to the maturation of the immune system (Martín et al., 2003; Fernández et al., 2013; Jeurink et al., 2013). The manipulation involved in DHM extraction and management may be an exogenous source of microbial contamination to DHM. In fact, contaminated human milk has occasionally been a vehicle for pathogenic microorganism, to preterm and very low birth weight infants causing late onset sepsis (Widger et al., 2010; Lanzieri et al., 2013; Kayiran et al., 2014; Nakamura et al., 2016).

To ensure microbiological safety, DHM is pasteurized in most HMBs in order to kill all non-spore forming and potentially pathogenic microorganisms (Landers and Updegrove, 2010). At present, a Low-Temperature Long-Time (heating at 62.5°C for 30 min) pasteurization, also known as “Holder” pasteurization (HoP), is the heat treatment most commonly applied to DHM. High-risk viruses, such as cytomegalovirus, papillomaviruses, and human immunodeficiency, T-lymphotrophic, Ebola, Marburg, and Zika viruses, are also destroyed after HoP of human milk (Orloff et al., 1993; Chiavarini et al., 2011; Rigourd et al., 2011; Donalisio et al., 2014; Hamilton Spence et al., 2017; Pfaender et al., 2017) while spore-forming bacteria, such as *Bacillus* sp., may survive this heating process (de Segura et al., 2012). On the other hand, some nutritional and biological components, such as immunoglobulins, hormones or enzymes, result negatively affected after HoP (Peila et al., 2016). Another disadvantage associated with the available commercial units for HoP is that the volume of production in a working day is limited to the number of containers with DHM that can be placed in the equipment.

Recently, the High-Temperature Short-time (HTST) pasteurization, a well-established heat treatment in dairy industry, has been proposed for the treatment of DHM (Peila et al., 2017; Picaud and Buffin, 2017). This method involves heating milk rapidly to 72°C, keeping it for a few seconds (usually 15 s), and cooling it down immediately. HTST is, at least, equivalent to HoP in terms of ensuring milk microbiological safety (Terpstra et al., 2007; Goelz et al., 2009) while is better at preserving the functionality of its biologically active components (Silvestre et al., 2008; Baro et al., 2011; Giribaldi et al., 2016).

Different experimental systems, such as laboratory capillary heat exchangers, industrial heat exchangers or benchtop pasteurizers for small volumes, have been used for HTST pasteurization of DHM (Goldblum et al., 1984; Dhar et al., 1996; Giribaldi et al., 2016) but they have never been tested in actual HMB conditions. Most HMB around the world are located within or close to local hospital NICUs, and often they do not provide enough DHM to meet all the existing demand, sometimes not even to feed the most vulnerable preterm infant's at the NICUs (DeMarchis et al., 2017). Centralization of DHM processing using a continuous HTST system and its distribution would expand notably the access to DHM, improving significantly infant health outcomes.

Alternative treatments to DHM thermal processing, including high pressure processing or UV irradiation, have been proposed. These alternatives seem to better preserve some biological components, such as total immunoglobulin A or lysozyme activity, compared to HoP (Christen et al., 2013; Mayayo et al., 2016; Sousa et al., 2016). However, they require further investigation about the influence on other milk components as well as performing exhaustive microbiological analyses before they could be used to treat DHM.

In this context, the objective of this work was to design a continuous HTST system to pasteurize DHM in the HMB operating environment and to evaluate its effect on the microbiological quality of DHM when tested at different temperatures and holding times. Alkaline phosphatase (ALP), γ-glutamyl transpeptidase (GGTP) activities and lactulose (4-O-β-D-galactopyranosyl-D-fructose) were analyzed in order to check their usefulness as heating indicators, while the potential nutritional damage was evaluated using furosine (ε-N-2-furoylmethyl-L-lysine) as indicator of the Maillard reaction, one of the main undesirable consequences of heat treatment.

## Materials and methods

### Human milk samples

DHM samples were obtained from the Regional Human Milk Bank “Aladina-MGU” located at the Hospital Universitario 12 de Octubre (Madrid, Spain). Milk collection was performed following a specific protocol for donor mothers approved by the Hospital 12 de Octubre Clinical Research Ethics Committe (ethical approval code: 12/325) and informed consent was obtained from each donor in accordance with the Declaration of Helsinki. Milk was collected at home using either electric (Lactaline; Ameda, Lincolnshire, Illinois, USA) or manual (Harmony or Lactaset models; Medela, Baar, Switzerland) pumps, frozen (−18°C) in a domestic refrigerator, and transported to the HMB in an insulated box provided with ice packs.

### Experimental design

**Figure 1:**
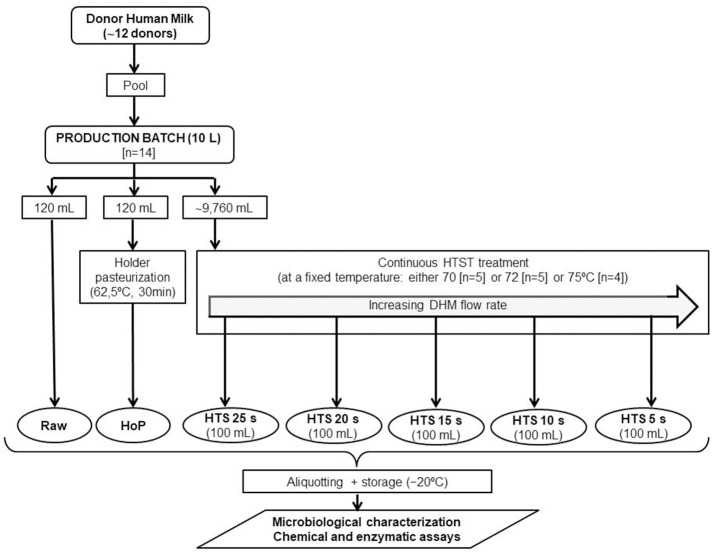
Experimental design of this study.

DHM samples were stored frozen (−20°C) until processing at the HMB and, then, thawed in a shaking water bath (Jeio Tech BS-21; Lab Companion, Seoul, Korea) at 37°C. Milk from multiple donors (~12 donors) was pooled in sterile 2-L Pyrex bottles in a vertical laminar-flow cabinet (Fortuna 1200 Maxi; Controltecnica Instrumentación Científica SL, Madrid, Spain). The content of these bottles was mixed to achieve a final volume of about 10 L (a production batch) in the stirred feed vessel of the HTST equipment (Figure 1). Then, the pH value of the production batch was measured and only batches having pH ≥ 6.5 were further processed (Escuder-Vieco et al., 2016). Two DHM aliquots (~120 mL) from each production batch were separated and transferred to 150-mL sterile glass containers (Nuk, Roche Diagnostics, S.L., Spain): one aliquot was not heat-treated, while the other one was subjected to standard HoP in the Regional HMB “Aladina-MGU” facilities. The rest of the batch (~9,760 mL) was HTST-processed at a fixed temperature (70 or 72 or 75°C) and different times (5, 10, 15, 20 and 25 s) using the new equipment. All aliquots of untreated (raw) or heat-processed DHM were stored frozen (−20°C) until analysis of microbial load, selected enzymatic activities, and chemical indicators of heat treatment (Figure 1).

### HTST processing of DHM

A continuous flow HTST system (Escuder-Vieco et al., 2017) was designed and constructed to process DHM at the facilities of the Regional HMB “Aladina-MGU” in collaboration with SIVE Fluid Systems (Alcalá de Henares, Madrid, Spain), a company specializing in fluid food processing.

The length of the holding tube (internal diameter of 9.4 mm) was calculated considering a flow rate of 10 L/h and a residence time of 15 s. The validation of the holding tube was done by operating the pasteurizer with water, injecting a saturated salt solution in the holding tube, and measuring the time required for the injection to traverse the tube (Food and Drug Administration, 2015)^1^. All measurements of residence time were performed in triplicate.

In this study, a total of 14 DHM production batches were HTST-processed at different temperatures: 5 batches at 70°C, 5 batches at 72°C, and 4 batches at 75°C. In order to obtain DHM samples that had been heated at a fixed temperature (either 70 or 72 or 75°C) and different residence time at the holding section (5–25 s), DHM flow rate was adjusted (Supplementary Table 1). To avoid microbiological contamination, DHM processing always started using the slowest flow rate, corresponding to the most stringent processing condition (25 s), and increased sequentially in order to obtain samples subjected to shorter processing times. Therefore, processing of one production batch delivered five DHM samples treated at one fixed temperature and five different processing times (5, 10, 15, 20, and 25 s). These samples (~100 mL) were taken sequentially in sterile glass containers at the exit of the closed isothermal tank.

Measurement of the refractive index to follow the concentration of total soluble solids (°Brix) of the milk during heat processing was done using a manual refractometer (RHB 32 ATC; Lumen Optical Instrument Co., Ltd., Fuzhou City, China).

### HoP of DHM

A 120 mL aliquot of DHM from each production batch (*N* = 14) was placed onto a sterile glass container and pasteurized using the Holder method (62.5°C for 30 min) in a shaking (30 rpm) water bath (Jeio Tech BS-21, Lab Companion, Oxfordshire, UK) provided with temperature control. After pasteurization, the containers were transferred immediately into another shaking water bath (Jeio Tech BS-21) filled with ice-cold water for fast cooling. Once the target temperature (4°C) was reached (always within the first 15 min of cooling), the milk was stored at −20°C until analysis. A wireless temperature data logger thermometer (Mesa Labs, Inc., Lakewood, Colorado, USA) was introduced in a control bottle (cow's milk) to be used as a probe to monitor the temperature of the milk batch by using the DataTrace Pro software (Mesa Labs). The water bath maintained the required temperature with a precision of ±0.2°C.

### Bacterial cultures, identification, and genotyping of the isolates

Proper peptone water dilutions of raw and heat-processed (either by Holder or HTST methods) DHM samples were spread onto Brain Heart Infusion (BHI, Oxoid, Basingstoke, UK; a general-purpose medium suitable for the cultivation of nonfastidious bacteria, yeasts, and molds), Columbia Nalidixic Acid (CNA, BioMérieux, Marcy l'Etoile, France; a highly nutritious, general-purpose medium for the isolation and cultivation of fastidious microorganisms from clinical samples, in particular Gram-positive bacteria such as *Staphylococcus, Streptococcus, Enterococcus*, and *Corynebacterium*), Kanamycin Aesculin Azide (KAA, Oxoid; a selective medium for the isolation of *Enterococcus*), and MacConkey (MCK, BioMérieux; a selective medium for the isolation of enterobacteria) agar media. Samples of hot water (100 μL) obtained immediately before starting the heat treatment of DHM were also plated onto the media listed above. Plates were aerobically incubated at 37°C for 24 to 48 h.

After bacterial counting, at least one bacterial colony showing a characteristic morphology (color, size, shape, halos) of each of the types present on every plate was selected for identification. All isolates were grown in BHI broth and stored at −80°C after the addition of glycerol (30%, v/v). Identification of the isolates was performed by matrix-assisted laser desorption/ionization time-of-flight mass spectrometry (MALDI-TOF MS) using the Vitek-MS™ system (BioMérieux) (Probisearch, Tres Cantos, Madrid, Spain). The isolates were identified at the species level when there was a 99 to 100% match to the species-specific *m/z* values in the database.

Genetic relatedness among selected enterococci isolated from unheated and heat-processed DHM was investigated by Random Amplification of Polymorphic DNA (RAPD). RAPD profiles were obtained using primer OPL5 (5′-ACGCAGGCAC-3′) that was originally designed for lactobacilli but it has been shown to be useful also for typing other bacterial groups (Veyrat et al., 1999; Ruiz-Barba et al., 2005). Isolates showing the same RAPD profile were subjected to Pulsed-Field Gel Electrophoresis (PFGE) in a CHEF DR II apparatus (Bio-Rad, Birmingham, UK) using the protocol described by Jiménez et al. (2008) except that DNA fragments were separated at a constant voltage of 200 V with a pulse time from 2 to 28 s for 24 h and, then, another from 15 to 60 s for 13 h. Computer-assisted analysis was performed with InfoQuest FP software (Bio-Rad Laboratories, Inc., Hercules, California, USA).

### Enzymatic assays

Alkaline phosphatase (ALP) activity was analyzed using the enzymatic Alkaline Phosphatase Diethanolamine Detection Kit (Sigma-Aldrich) and adapted to a 96-well plate format. This assay uses *p*-nitrophenyl phosphate (*p*NPP) as the substrate. The enzymatic hydrolysis of *p*NPP to *p*-nitrophenol (*p*NP) and inorganic phosphate at 37°C was followed for 2 h by monitoring the increase in absorbance at 405 nm (*p*NP) at pH 9.8 using a microplate spectrophotometer (Zenyth 200RT, Anthos Labtec Instruments GmbH, Saltzburg, Austria). All DHM samples (stored at −20°C) were thawed on ice and diluted to 1:5 (v/v) in distilled water before assayed as described by the manufacturer. The activity of ALP was expressed as nmoles of *p*NP released per mL of DHM and per minute under the assay conditions.

γ-glutamil transpeptidase (GGTP) activity was assayed spectrophotometrically using γ-glutamyl-*p*-nitroanilide (Sigma) and glycylglycine (Sigma) as substrates as described by McKellar et al. in a 96-well plate format (McKellar et al., 1991). In this assay, GGTP catalyzes the synthesis of the tripeptide γ-glutamylglycilglycine and the release of *p*-nitroaniline at 37°C for 20 min. The activity was determined after a diazo coupling reaction with the released *p*-nitroaniline by monitoring the increase in absorbance at 540 nm using a microplate spectrophotometer (Zenyth 200RT). The activity of GGTP was expressed as μmol of *p*NA released per mL of DHM and per minute under the assay conditions. All samples were diluted 1:50 (v/v) in distilled water, except Holder pasteurized DHM samples that were diluted 1:20 (v/v).

### Determination of furosine and lactulose

Furosine analysis in raw and heat-treated DHM samples was carried out by ion-pair reverse-phase high-performance liquid chromatography (RP-HPLC) following the method of Resmini et al. (Resmini et al., 1990), although it was adapted to smaller volumes (1 mL) as described by Espinosa-Martos et al. (2013). Protein concentrations were determined by the bicinchoninic acid (BCA) assay using the BCA Protein Assay Reagent (Thermo Fisher Scientific Inc., Rockford, IL) and bovine serum albumin as standard. All analyses were done in duplicate, and the values were expressed as milligrams of furosine per 100 g of protein.

Lactulose concentration in raw and heat-treated DHM samples was determined by gas chromatography with flame ionization detector, following the method described by Montilla et al. (2005) with the modifications described by Espinosa-Martos et al. (2013). The identity of lactulose present in the samples was confirmed by comparison with relative retention times of a standard. Quantitative analysis was carried out by the internal standard method. Response factors were calculated after triplicate analysis of lactulose standards (1–50 mg/L). The limit of detection and the limit of quantification of the gas chromatography method for lactulose were 4 and 10 mg/L, respectively.

### Statistical analysis

Microbial counts were recorded as colony-forming units (cfu/mL) and were transformed to log_10_ values before statistical analysis. Results are displayed as means and 95% confidence interval (CI) of the mean or as means ± standard deviation (SD). The significance of differences between means was assessed using one-way ANOVA and Tukey multiple comparison *post-hoc* tests or paired Student's *t*-tests. The level of significance was set at *p* < 0.05. The analyses were performed using the StatGraphics Centurion XVII version 17.0.16 (Statpoint Technologies, Inc., Warrenton, VA, USA). Cluster analysis of RAPD and PFGE pattern profiles was performed using the UPGMA method based on the Dice correlation similarity coefficient and the InfoQuest FP software (Bio-Rad).

## Results

### Design of the HTST system

**Figure 2:**
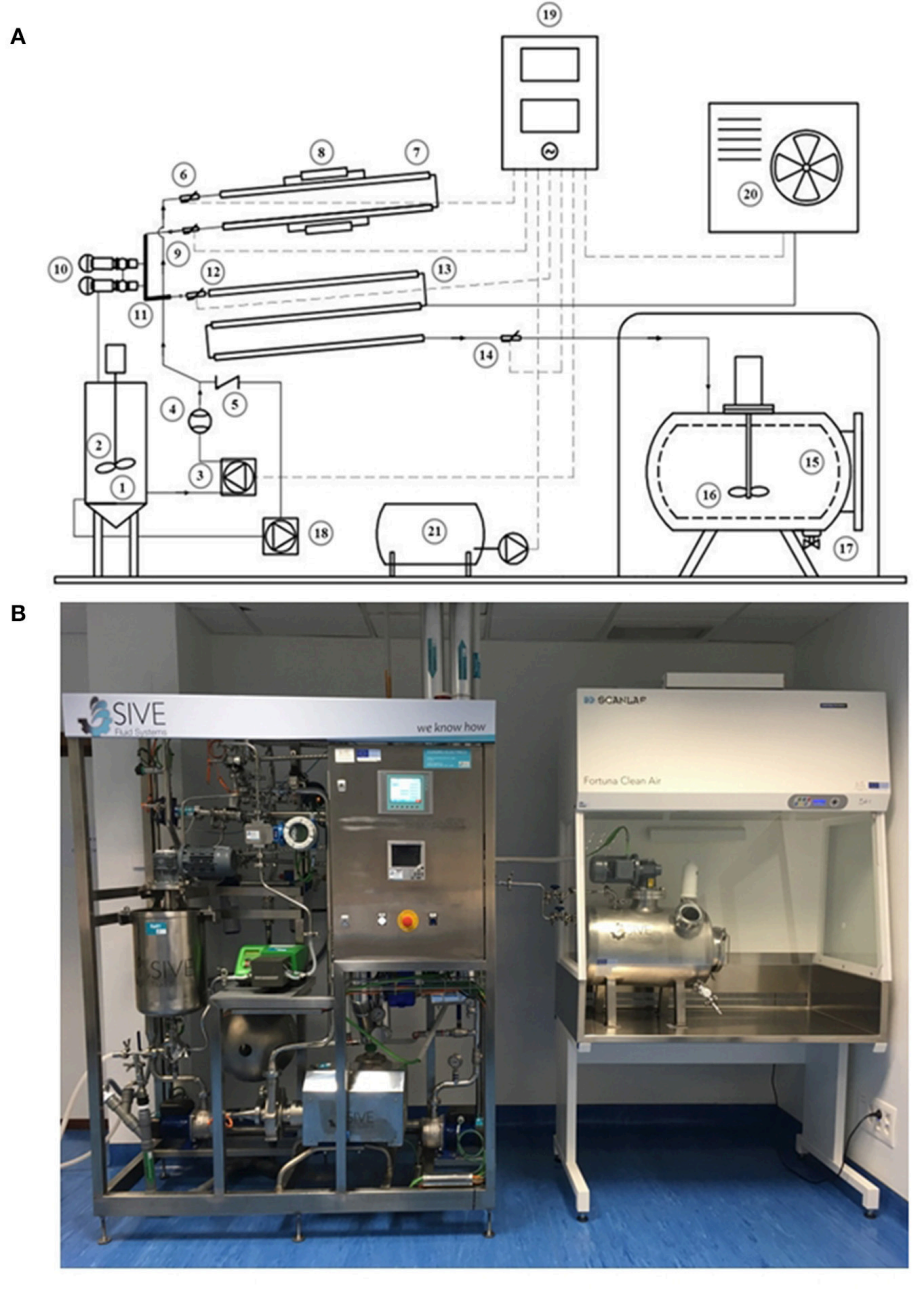
HTST system developed to process DHM. **(A)** Schematic diagram of the HTST equipment showing its basic components and milk flow. 1: Feed vessel for raw milk; 2: Stirring system; 3: Peristaltic pump; 4: Flowmeter; 5: Backflow valve; 6: Temperature probe; 7: Heating section; 8: Thermal resistances; 9: Temperature probe; 10: Safety valves; 11: Holding tube; 12: Temperature probe; 13: Cooling section; 14: Temperature probe; 15: Closed isothermal tank for pasteurized milk; 16: Stirring system; 17: Filling system; 18: Secondary pump; 19: Control unit; 20: Cold generation equipment; 21: Air compressor. **(B)** Equipment for the HTST treatment of donor milk at the Regional HMB “Aladina-MGU”.

The HTST system consists of the following major units: holding tanks and pumps, heat exchange and holding sections, temperature and flow controllers, and a recording device (Figures 2A, B). All parts of the equipment that come in contact with DHM were made of AISI Type 316 stainless steel.

The open stirred feed vessel (20 L) that provides a constant supply of milk was equipped with an impeller driven by a DC motor, used at low speed (90–95 rpm), to mix the pooled DHM and avoid phase separation during the process. A tuning fork switch controls the volume of milk present in this vessel and also allows the recirculation of fluid, if required. A peristaltic pump conveys the fluid (DHM or water) from the feed vessel to the heat exchangers under pressure at a flow rate of 10 L/h. The flow rate of the solution is regulated by adjusting the rotor disc speed and is measured by an electromagnetic flow meter.

The core of the HTST system includes three sections: heating, holding and cooling (Figure 2A). The heating and cooling sections are corrugated mono-tube heat exchangers that are installed in series and arranged into two separate sections. The first section comprises two heat exchangers to preheat DHM with hot water to the desired processing temperature (either 70 or 72 or 75°C in this work). The second section is made up of other three heat exchangers to quickly cool down the heat-treated DHM to 2–4°C with chilled water. The monotube configuration of the heat exchangers determines that the milk flows through the inner tube while the hot or cold water flows in a counter-current arrangement through the annular space between the inner and the outer tube. The holding tube (0.648 m), that ensures the proper time of treatment at the specified temperature, is located between the heating and the cooling sections. Sizing of the holding tube was validated by the salt conductivity test. The average of three measurements was 15.27 ± 0.11 s and the individual measurements were within 0.3 s of each other.

Four temperature transmitters measure the fluid temperature before entering and after leaving the heating section, after leaving the holding tube, and after leaving the cooling section. A pressure transmitter monitors continuously the pressure of heat-treated milk at the exit point. Two independent secondary circuits provide hot and cold water for the process.

A closed isothermal tank (25 L) with an impeller driven by a direct current motor and used at low speed (90–95 rpm) holds the pasteurized milk (Figure 2A). This tank allows separating milk packaging from heat processing and is located in a laminar flow safety cabinet provided with a filter (0.22 μm) to prevent microorganisms and particles entering the deposit. The tank has a manual valve for dispensing the required volume of pasteurized DHM in sterile containers, according to the demand of the HMB.

The equipment also has a control unit composed of a touch screen display to adjust all operating parameters and procedures and a recorder to gather the records of all parameters (such as milk flow rate, temperature of the fluid at several points of the process, and temperature of the hot water circuit) (Figure 2A). All acquired data were downloaded and analyzed with dedicated software in order to assess the adequacy of the process.

### Operation of the HTST system

**Figure 3:**
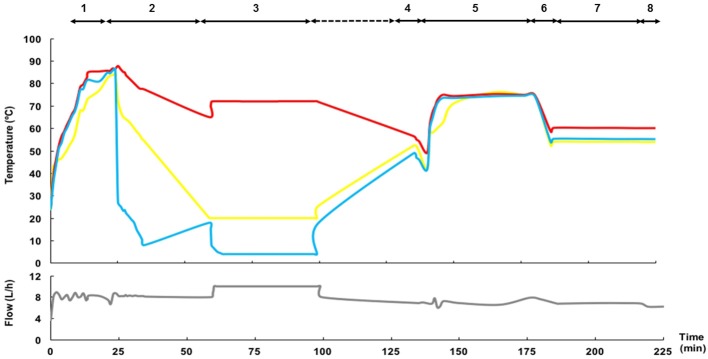
Time-temperature and fluid flow profiles of a representative HTST process. The main steps of operation are: 1: Sanitizing; 2: Conditioning; 3: Heat processing and cooling. The inlet temperature in the heating section (either hot water in steps 1 and 2, raw DHM in step 3, or water or cleansing solutions in steps 4–8) is shown in yellow; the temperature of the fluid at the exit of the holding tube is shown in red; and, the temperature of the fluid at the exit of the cooling section is shown in blue. The gray line at the bottom represents the fluid flow. The steps of the Cleaning-In-Place (CIP) procedure have been also included (4: Pre-rinse; 5: Alkaline wash; 6: Rinse; 7: Acid wash; 8: Final rinse).

Prior to each trial, the equipment was sanitized using circulating hot water (85°C) for at least 5 min for proper thermal sanitizing (Figure 3). Next, the temperature of processing (for both the heating and cooling sections) was equilibrated at a water flow rate of 10 L/h to reach the operating conditions. Then, DHM (10 L) was poured into the feed vessel and mixed for ~5 min. Milk processing started after checking the pH of the production batch was >6.5. The first portion of the flowing liquid (consisting in a mixture of water and DHM) leaving both the heating section (through the return to the feed vessel relief valve; ~120 mL) and the cooling section (through the aseptic closed tank; ~120 mL) was discarded until the refractive index of the fluid was ~9.5 °Brix. A typical profile of fluid temperature and flow during the operation of the HTST unit is shown in Figure 3.

The HTST system incorporates a Cleaning-In-Place (CIP) system. The cleaning procedure comprises four steps after each processing. First, a pre-rinse step is done immediately after each production by recirculation of hot water (50°C) for at least 5 min to eliminate all traces of milk from the system (Figure 3). The second step is an alkaline wash using a detergent with a sodium hypochlorite basis (SI-20, SIVE Fluid Systems) at 75°C for 30 min, followed by a rinsing cycle (50°C, 5 min). The third step is an acid wash using a cleaner based on hydrochloric acid (SI-40, SIVE Fluid Systems) at 50°C for 30 min, followed by a rinsing round (50°C, 5 min) until the rinsing water has a pH value of 6 to ensure that all traces of acid cleaner have been removed.

The effectiveness of the thermal sanitizing step was checked for each processing cycle by microbiological analysis.

### Microbiological performance testing

**Figure 4:**
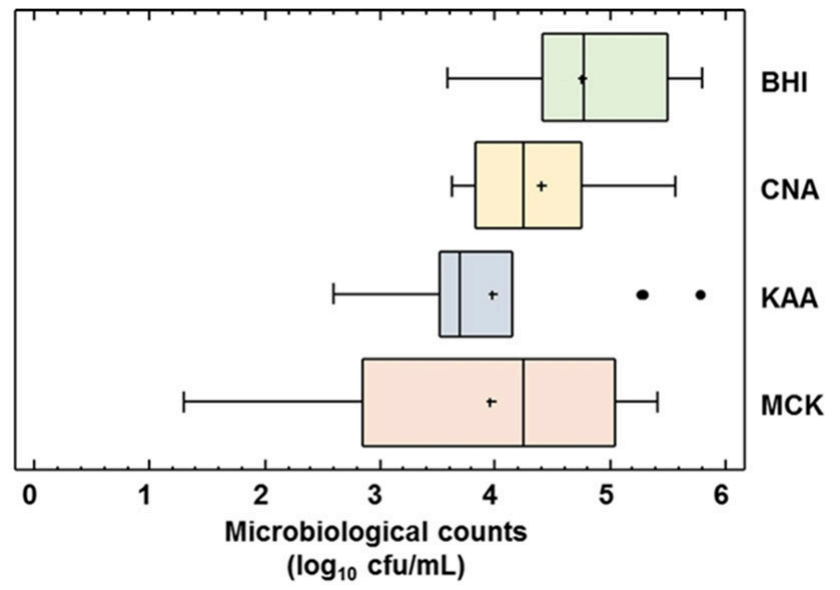
Microbiological counts in raw DHM. Total microbiological counts (of all the raw DHM batches (*N* = 14) used for heat processing trials in BHI, Brain Hearth Infusion; CNA, Columbia Nalidixic Acid; KAA, Kanamycin Aesculin Azide; MCK, MacConkey agar plates were expressed as log_10_ cfu/mL. The central rectangle represents the interquartile range (IQR), the line and the cross inside the rectangle show the median and the mean, respectively; the whiskers indicate the maximum and minimum values, and the black dots outside the rectangles are suspected outliers (>1.5 × IQR).

The global microbiological analysis of the raw DHM used for the 14 production batches revealed the presence of microorganisms in all the samples when cultured in any of the culture media (BHI, CNA, MCK, and KAA) employed in this study (Figure 4). Globally, the mean (95% CI) bacterial counts in DHM samples before the heat treatment oscillated between 3.96 (3.22–4.70) log_10_ cfu/mL in MCK and 4.76 (4.33–5.18) log_10_ cfu/mL in BHI (Figure 4). There was not a statistically difference in the mean values of the bacterial counts obtained in the different media (one-way ANOVA, *F* = 2.46, *p* = 0.073). Identification of the isolates obtained in the different growth media revealed their poor selectivity since some bacterial species could be isolated from up to three different growth media (e.g., *Enterococcus faecalis* and *Staphylococcus epidermidis* were isolated from BHI, CNA and KAA plates) (Supplementary Table 2).

The number of different microorganisms in a same raw DHM production batch ranged from 3 to 10, although seven was the most frequent number (Supplementary Table 2). In relation to Gram-positive bacteria, *S. epidermidis* and *E. faecalis* were present in all raw DHM batches (*N* = 14) with a mean count value of 4.16 and 3.97 log_10_ cfu/mL, respectively (Table 1). Other staphylococcal species such as *Staphylococcus aureus* (in 50% of the samples) and *Staphylococcus lugdunensis* (in 43% of the samples) were also isolated frequently but having slightly lower total viable counts (3.55 and 3.52 log_10_ cfu/mL, respectively).

**Table 1:** Microbiological characterization of the 14 batches of raw DHM analyzed in this study.

**Microorganism**		**Microbial counts**^b^		
	**n (%)^a^**	**Mean (95% CI)**	**Minimum**	**Maximum**
**GRAM-POSITIVE BACTERIA**				
*Bacillus cereus*	1 (7)	2.45		
*Clostridium ramosum*	1 (7)	5.25		
*Corynebacterium tuberculostearicum*	1 (7)	3.20		
*Enterococcus faecalis*	14 (100)	3.97 (3.50–4.45)	2.77	5.77
*Staphylococcus aureus*	7 (50)	3.55 (2.98–4.11)	2.88	4.50
*Staphylococcus epidermidis*	14 (100)	4.16 (3.87–4.44)	3.27	4.90
*Staphylococcus lugdunensis*	6 (43)	3.52 (2.50–4.53)	1.88	4.55
**GRAM-NEGATIVE BACTERIA**				
*Acinetobacter baumanni* complex	3 (21)	3.45 (1.04–5.85)	2.34	4.11
*Acinetobacter ursingii*	5 (36)	3.25 (2.18–4.33)	2.30	4.00
*Chryseobacterium indologenes*	3 (21)	3.58 (2.95–4.22)	3.30	3.77
*Enterobacter asburiae/cloacae*	5 (36)	2.78 (1.85–3.70)	1.69	3.61
*Enterobacter gergoviae*	1 (7)	2.99		
*Klebsiella oxytoca*	7 (50)	3.12 (2.34–3.90)	1.84	4.17
*Klebsiella pneumoniae*	4 (29)	2.79 (1.33–4.24)	1.84	4.03
*Pantoea aglomerans*	1 (7)	2.34		
*Pseudomonas aeruginosa*	3 (21)	5.10 (4.52–5.69)	4.92	5.37
*Pseudomonas fluorescens*	3 (21)	4.78 (2.70–6.86)	4.30	5.75
*Pseudomonas putida*	2 (14)	3.89	2.88	4.90
*Serratia marcescens*	2 (14)	3.51	3.00	4.03
*Serratia grimessi*	1 (7)	4.53		
*Serratia liquefaciens*	1 (7)	2.75		
*Stenotrophomonas maltophilia*	4 (29)	4.18 (2.51–5.86)	2.77	5.30
**YEASTS**				
*Candida parapsilosis*	3 (21)	3.38 (1.57–5.19)	2.69	4.14
*Trichosporon asahii*	1 (7)	1.69		

a *Number (%) of DHM batches where the species was detected*.

b *Microbial counts obtained in all culture media were expressed as log_10_ cfu/mL. Supplementary Table 2 details the culture media where each microorganism could be isolated*.

*CI, confidence interval of the mean*.

The variety of Gram-negative bacteria identified was wider than that of Gram-positive ones and, globally, included isolates belonging to eight genera (Table 1, Supplementary Table 2). The species most frequently found in this group was *Klebsiella oxytoca* (in 50% of the raw DHM batches), followed by *Acinetobacter ursingii* (36%) and members of the *Enterobacter cloacae* complex (36%) (Table 1). Although the frequency of detection of some species of *Pseudomonas* and *Stenotrophomonas* in raw DHM was lower, such bacteria reached the highest levels (5.10 and 4.78 log_10_ cfu/mL for *Pseudomonas aeruginosa* and *Pseudomonas fluorescens*, respectively) in the batches where they could be isolated from. Yeasts were scarcely isolated (Table 1).

The efficacy of the HTST system regarding microbial inactivation was determined at 70, 72, and 75°C using holding times ranging from 5 to 25 s. Survivors were detected only in five production batches and were identified as *B. cereus* and *E. faecalis* (Table 2). *B. cereus* was recovered at mean levels ranging from 1.58 to 2.57 log_10_ cfu/mL in five production batches (two batches from treatment at 70°C, two at 72°C and one at 75°C), independently of the holding time (Table 2). *E. faecalis* was isolated in three production batches after processing at 70°C and, also, in one batch after processing at 72°C for 5 s (KAA plate), but it was not recovered when this temperature (72°C) was held for longer time (>5 s) (Table 2). Bacterial growth was not detected in any MCK plate (the detection limit of the method was ~1.30 log_10_ cfu/mL), indicating the destruction of all Gram-negative bacteria even at the lowest temperature and the shortest time for all the heat treatments investigated in this work (Supplementary Table 3).

**Table 2:** Bacterial identification of survivors recovered after HTST and HoP treatments of the 14 DHM batches.

**Heat treatment**	**n/N^a^**	**Microorganism**	**Growth medium^b^**	**Total counts^c^ (mean ± SD)**
HTST 70°C, 5 s	1/5	*B. cereus*	BHI, CNA	1.79 ± 0.69
	3/5	*E. faecalis*	BHI, CNA, KAA	2.13 ± 0.54
HTST 70°C, 10 s	1/5	*B. cereus*	BHI	2.20
	2/5	*E. faecalis*	BHI, CNA, KAA	2.16 ± 0.80
HTST 70°C, 15 s	2/5	*B. cereus*	BHI	1.58 ± 0.83
	2/5	*E. faecalis*	BHI, CNA, KAA	1.74 ± 0.53
HTST 70°C, 20 s	1/5	*B. cereus*	BHI	2.14
	2/5	*E. faecalis*	BHI, CNA	1.91 ± 0.29
HTST 70°C, 25 s	1/5	*B. cereus*	BHI	2.04
	1/5	*E. faecalis*	BHI, CNA	2.22 ± 0.11
HTST 72°C, 5 s	2/5	*B. cereus*	BHI	2.27 ± 0.03
	1/5	*E. faecalis*	KAA	1.30
HTST 72°C, 10 s	2/5	*B. cereus*	BHI	2.40 ± 0.14
HTST 72°C, 15 s	2/5	*B. cereus*	BHI	2.43 ± 0.12
HTST 72°C, 20 s	2/5	*B. cereus*	BHI	2.55 ± 0.03
HTST 72°C, 25 s	2/5	*B. cereus*	BHI	2.57 ± 0.00
HTST 75°C, 5 s	1/4	*B. cereus*	BHI	2.23
HTST 75°C, 10 s	1/4	*B. cereus*	BHI	2.20
HTST 75°C, 15 s	1/4	*B. cereus*	BHI	1.95
HTST 75°C, 20 s	1/4	*B. cereus*	BHI	2.07
HTST 75°C, 25 s	1/4	*B. cereus*	BHI	2.17
HoP (62.5°C, 30 min)	5/14	*B. cereus*	BHI,CNA	2.06 ± 0.44

a *n/N, number of DHM batches where microbial counts were enumerated by plate counting (n) out of the total batches processed at that temperature (N)*.

b *Growth media where the isolates were recovered: BHI, Brain Hearth Infusion; CNA, Columbia Nalidixic Agar; KAA, Kanamycin Aesculin Agar*.

c *Total microbiological counts obtained in all culture media (log_10_ cfu/mL). Supplementary Table 3 details the total counts for each culture media*.

*SD, standard deviation*.

**Figure 5:**
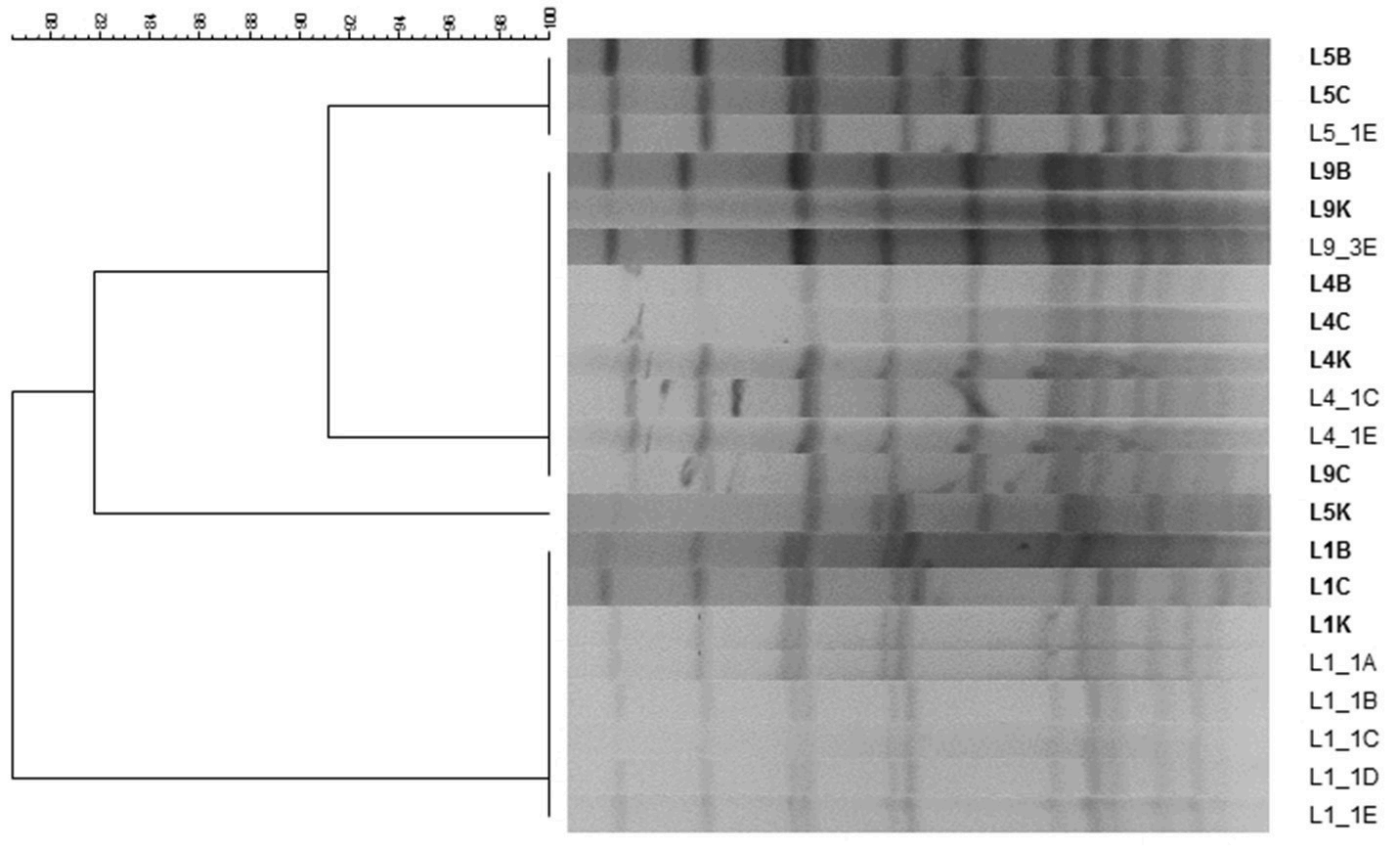
PFGE profiles of enterococci isolated from 4 production batches of DHM before and after several HTST treatments. B, isolate recovered from Brain Hearth Infusion (BHI); C, isolate recovered from Columbia Nalidixic Acid (CNA); K, isolate recovered from Kanamycin Aesculin Azide (KAA) agar plates. L1B, L1C, L1K, L4B, L4C, L4K, L5B, L5C, L5K, L9B, L9C, and L9K were isolated from raw DHM samples, while L1_1A, L1_1B, L1_1C, L1_1D, L1_1E, L4_1C, L4_1E, L5_1E, and L9_3E were isolated from HTST-processed DHM samples.

The analysis of PFGE profiles of the enterococci isolated from DHM samples before and after different heat treatments revealed the existence of, at least, four pulsotypes that were closely associated to specific production batches (Figure 5). In fact, as an example, the same pulsotype was shared by *E. faecalis* isolates recovered from milk samples taken from the same production batch before (L1C, L1B, L1K) and after (L1_1A, L1_1B, L1_1C, L1_1D, L1_1E) the heat treatment. Isolates from different batches did not share the same PGFE profile, except for two production batches (L4 and L9) where the same profile was shared by all nine isolates. In one production batch (L5), two different PFGE profiles were found before heat treatment (L5B/L5C and L5K) but only one was recovered after the heat treatment (L5_1E = L5B/L5C).

Bacterial survival after HoP of DHM samples (*N* = 14) was observed in 5 batches when pasteurized milk samples were cultured on BHI and CNA agar plates (2.06 ± 0.44 log_10_ cfu/mL) (Supplementary Table 3). In all of these cases, *B. cereus* was the microorganism isolated (Table 2). No other microorganism was detected neither in BHI nor in CNA, KAA or MCK plates after HoP, indicating the inactivation of the rest of the microorganisms that were present initially in pooled raw DHM.

All hot water samples collected at the end of the sanitation step, and prior the processing of a DHM production batch, over the course of this study were seeded on BHI agar for microbiological analysis. Bacterial growth was not observed in any sample.

### Enzymatic markers of heat-treatment in DHM

**Figure 6:**
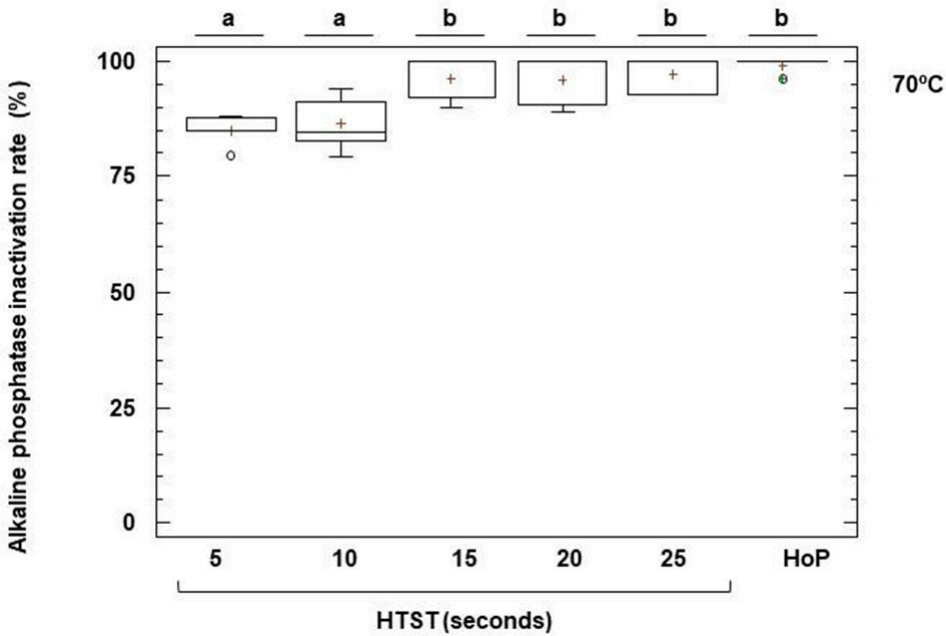
Inactivation of alkaline phosphatase (ALP) activity after HTST and HoP treatments. HTST treatments were performed at 70 for 5, 10, 15, 20, and 25 s in 5 DHM batches and Holder pasteurization (HoP) at 62.5°C for 30 min in 14 DHM batches. The central rectangle represents the interquartile range (IQR), the line and the cross inside the rectangle shows the median and the mean, respectively, the whiskers indicate the maximum and minimum values, and the dots outside the rectangles are suspected outliers (>1.5 × IQR). The letters above the boxplots indicate statistically significant differences in the inactivation rate of ALP at 70°C (one-way ANOVA, *F* = 8.72, *p* < 0.0001).

The activities of ALP and GGTP, two enzymes naturally present in milk, can be used as indicators of the intensity of a heat treatment. Unheated DHM exhibited a mean (95% CI) value of ALP activity of 115.96 (89.20–142.72) nmoles of *p*NP/mL min (Figure 6). This enzyme was completely inactivated after HoP of DHM (Figure 6). In DHM heated at 70°C there was a small amount of remaining activity, ranging from 15% after 5 s to 3% after 25 s (Figure 6), but the enzyme was completely destroyed at 72 and 75°C, even at the shortest time of treatment (5 s).

**Figure 7:**
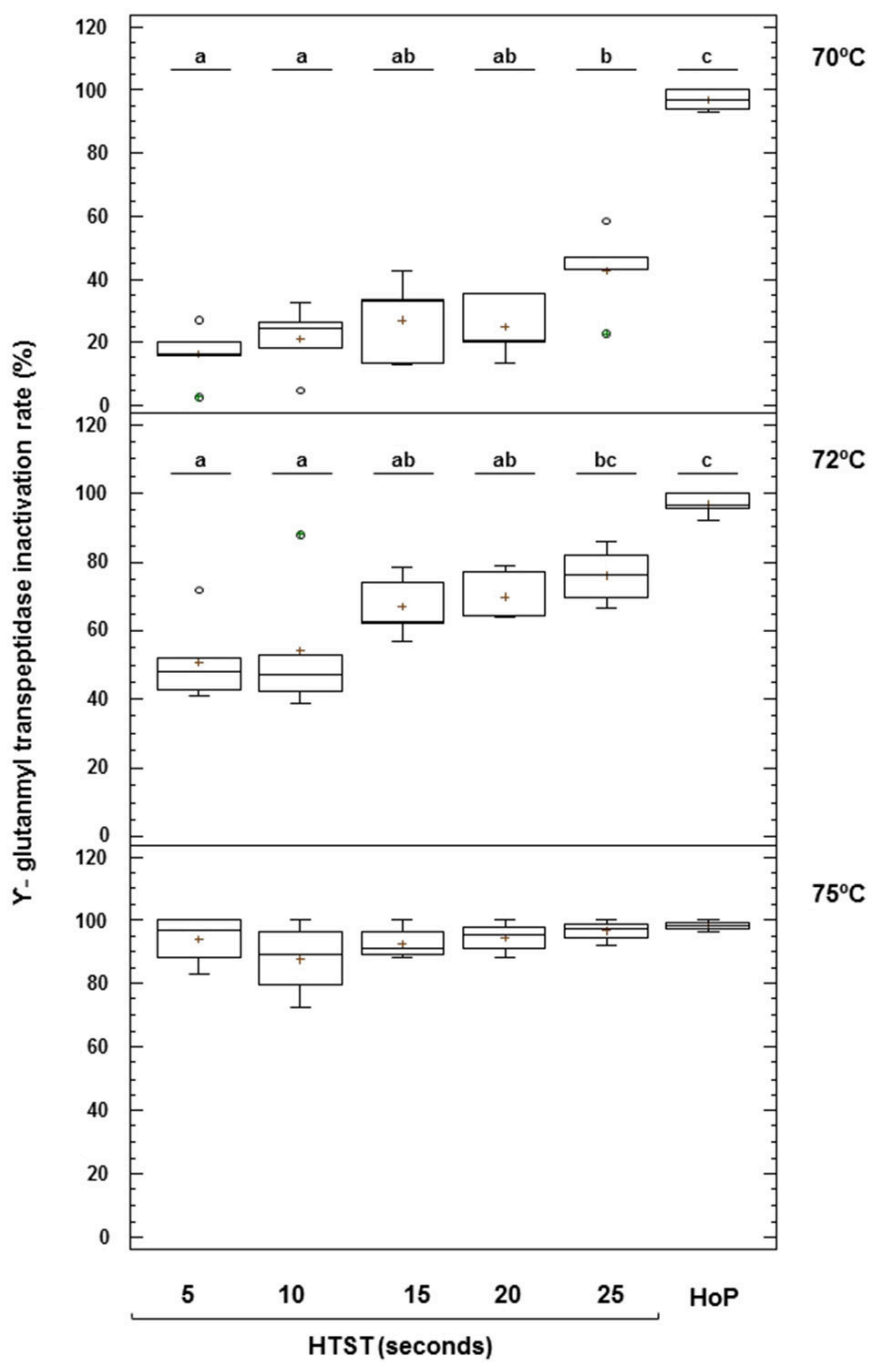
Inactivation of γ-glutamyl transpeptidase (GGTP) activity after HTST and HoP treatments. HTST treatments were performed at 70, 72, and 75°C for 5, 10, 15, 20, and 25 s and Holder pasteurization (HoP) at 62.5°C for 30 min in 14 DHM batches (5 batches at 70°C, 5 batches at 72°C, and 4 batches at 75°C). The central rectangle represents the interquartile range (IQR), the line and the cross inside the rectangle shows the median and the mean, respectively, the whiskers indicate the maximum and minimum values, and the dots outside the rectangles are suspected outliers (>1.5 × IQR). The letters above the boxplots indicate statistically significant differences in the inactivation rate of GGTP at 70 and 72°C (one-way ANOVA, *F* = 42.44 at 70°C and *F* = 10.96 at 72°C, *p* < 0.000).

The mean (95% CI) value of the activity of GGTP in unheated DHM was 2.09 (1.70–2.50) μmol of *p*NA/mL min (Figure 7). This enzyme was also destroyed after HoP but its thermoresistance was higher than that of ALP (2–3% of remaining activity). Production batches of DHM heated at 70°C retained a significant amount of GGTP activity, ranging from 84% after 5 s to 57% after 25 s. HTST treatment caused a progressive destruction of this enzyme with increasing temperatures and holding times, although about 24% of activity could be still detected after heating at 72°C for 25 s. At 75°C, the enzyme was inactivated and, in fact, there was no significant difference between the remaining GGTP activity after HoP and after HTST treatments at 75°C (one-way ANOVA, *F* = 1.20, *p* = 0.348) (Figure 7).

### Chemical indicators of heat-treatment in DHM

Furosine was detected in all DHM samples analyzed, both before and after any of the applied heat treatments, and its content is presented in Table 3. Furosine levels in non-heated samples (*n* = 10) ranged from 1.3 to 2.5 mg/100 g protein, with a mean value of 2.1 mg/100 g protein. A clear trend toward increasing furosine concentrations with increasing temperature and time of treatment was noted, although the amount of furosine found in raw milk differed significantly from the amount found in HTST-treated DHM at 70°C for 15, 20 and 25 and at 72°C for 15 and 25 s (Table 3). Holder pasteurization of DHM increased significantly the concentration of furosine (mean value of 4.7 mg/100 g protein) in comparison with untreated DHM (paired Student *t*-test, *p* < 0.000).

**Table 3:** Effect of HTST and HoP treatments on the furosine concentration in DHM.

		**Furosine (mg/100 g of protein)**			
	**n^a^**	**Mean (95% CI)**	**Min**	**Max**	***p*-value^b^**
Raw	3	1.9^a^ (1.3–2.4)	1.3	2.3	0.002
HTST 70°C, 5 s	3	2.6^a^^b^ (2.0–3.1)	2.3	2.8	
HTST 70°C, 10 s	3	2.8^a^^b^^c^ (2.2–3.3)	2.6	3.1	
HTST 70°C, 15 s	3	3.1^b^^cd^ (2.6–3.7)	2.6	3.4	
HTST 70°C, 20 s	3	4.3^e^ (2.8–3.8)	3.8	4.9	
HTST 70°C, 25 s	3	3.6^cde^ (3.0–4.1)	3.2	4.3	
Holder (62.5°C, 30 min)	3	3.9^de^ (4.1–5.0)	3.2	4.9	
Raw	4	2.0^a^ (1.7–2.4)	1.8	2.3	0.001
HTST 72°C, 5 s	4	3.1^a^^b^ (2.7–3.5)	2.8	3.5	
HTST 72°C, 10 s	4	3.0^a^^b^ (2.6–3.5)	2.6	3.4	
HTST 72°C, 15 s	4	3.4^b^ (2.9–3.8)	2.9	4.1	
HTST 72°C, 20 s	4	3.2^a^^b^ (2.8–3.6)	2.7	3.5	
HTST 72°C, 25 s	4	3.3^b^ (2.9–3.7)	2.7	3.9	
Holder (62.5°C, 30 min)	4	5.2^c^ (4.8–5.6)	4.4	6.1	
Raw	3	2.3^a^ (1.8–2.8)	2.2	2.5	0.011
HTST 75°C, 5 s	3	3.3^a^ (2.8–3.9)	2.9	3.6	
HTST 75°C, 10 s	3	3.0^a^ (2.4–3.5)	2.6	3.3	
HTST 75°C, 15 s	3	3.2^a^ (2.7–3.7)	2.9	3.4	
HTST 75°C, 20 s	3	3.0^a^ (2.5–3.5)	2.8	3.1	
HTST 75°C, 25 s	3	3.7^a^^b^ (3.1–4.2)	3.1	4.6	
Holder (62.5°C, 30 min)	3	5.0^b^ (4.5–5.6)	3.8	5.8	

a *Number of DHM processing batches where furosine was analyzed*.

b *One-way ANOVA to compare furosine concentration in untreated (raw) DHM and after HTST treatments at different holding times and HoP. Superscript letters on mean values indicate statistical differences when Tukey's post-hoc pairwise comparison tests were done*.

*SD, standard deviation; CI, confidence interval*.

Lactulose was not detected neither in raw nor in HTST-treated DHM, but it was found in HoP DHM (*n* = 10). In fact, mean (95% CI) concentration of lactulose in nine samples was 22.22 (15.51–28.93) mg/L, while in one sample lactulose was present below the quantification limit of the method (10 mg/L).

## Discussion

In this study, the development of an HTST system specifically designed to fulfill the needs of a HMB for processing DHM is documented. This HTST system allows the fast and continuous pasteurization of variable amounts of DHM in controlled conditions. The heat exchangers were designed and constructed in order to have a self-draining design and a small dead volume (about 300 mL), thereby minimizing DHM losses. In addition, the heat treatment is independent of packaging, allowing packing pasteurized DHM in accordance with the needs and preferences of the HMB. CIP cleaning and sanitation of the whole HTST system before operation without dismantling the equipment and without direct manipulation of chemical products contributes to enhance safety and DHM quality.

At present, there are more than 650 HMBs, the most important providers of DHM to NICUs, around the world. More are planned to open in a close future to foster the use of DHM in preterm infants when MOM is not available or enough to cope with the neonate requirements (Haiden and Ziegler, 2016), following the recommendations of organisms such as the World Health Organization^2^, the European Society of Pediatric Gastroenterology, Hepatology and Nutrition (ESPGHAN Committee on Nutrition et al., 2013), the Human Milk Banking Association of North America (Human Milk Banking Association of North America, 2015), and the American Academy of Pediatrics (Section on breastfeeding American Academy of Paediatrics, 2012).

There has been some controversy regarding the use of raw or pasteurized DHM. In a few countries, such as Japan and Norway, there is a long tradition of administrating raw DHM having the advantage of providing all bioactive components including commensal bacteria, but it requires a particularly strict control and screening of donors (Grøvslien and Grønn, 2009; Mizuno et al., 2015). On the other hand, DHM is a vehicle for potentially pathogenic microorganisms and, as a consequence, most HMBs consider that DHM must be processed in order to guarantee its microbiological safety. In spite of undesirable changes related to heat treatment, pasteurized DHM still retains some beneficial and protective effects, and it is preferred to infant formula (Quigley and McGuire, 2014; Sisk et al., 2017). Unfortunately, the pasteurizers commercially available at present are very limited regarding the retention of some bioactive components and the amount of milk that can be processed (between 4 and 10 L per batch) (Underwood, 2013).

Bacteria could be isolated from all the raw human milk batches in the different culture media employed in this study. Most staphylococci, streptococci, corynebacteria, and enterococci may be associated to physiological human milk microbiota (Fernández et al., 2013). The high concentrations of enterobacteria, *Pseudomonas* spp. and other Gram-negative bacteria found in some of the samples may be explained by the fact that donors extracted the milk using pumps (Landers and Updegrove, 2010); milk extraction using these devices has been associated to high counts of such bacterial groups, which are not usual components of the human milk microbiota. Contaminating bacteria, arising from environment or rinsing water (Cervia et al., 2008; Fihman et al., 2012; Cohen et al., 2017) or being the result of poor hygienic practices (Boo et al., 2001; Brown et al., 2005), may persist in milk pumps and/or their accessories, even after application of current cleaning protocols that do not ensure their destruction (Marín et al., 2009).

In 2004, the National Advisory Committee on Microbiological Criteria for Foods (NACMC) defined pasteurization as “Any process, treatment, or combination thereof, that is applied to food to reduce the most resistant microorganism(s) of food health significance to a level that is not likely to present a public health risk under normal conditions of distribution and storage” (NACMCF, 2004)^3^. In dairy industry pasteurization is designed to achieve at least a five-log reduction (or 99.999% killing) of the most heat-resistant non-sporulating pathogen likely to be present in cow milk (*Coxiella burnettii*); this requires heating every particle of the milk to 72°C for 15 s or an equivalent treatment. Spore-forming bacteria, on the other hand, can survive milk pasteurization (Sarkar, 2015).

The purpose of pasteurization is making DHM safe for consumption, and this requires destroying all common pathogens (O'Connor et al., 2015). However, the most thermoresistant non-sporulated microorganism has not been defined in DHM. On the other hand, as the microbiological quality of pasteurized DHM depends on the initial microbiota present in raw DHM, the specific processing conditions applied, and the storage conditions, donors should receive a good education on hygienic milk extraction and storage from the HMB staff. In addition, HMB professionals should control environmental contamination during DHM management (Becker et al., 2016). In order to reach the pasteurization objective, this process should be implemented to heat the milk homogeneously, at a consistent temperature and for the correct time.

Only two bacterial species (*B. cereus* and *E. faecalis*) were able to survive (although at low concentrations) in some of the batches after the HTST treatments tested in this work. Holder pasteurization kills commensal and contaminant vegetative bacterial cells found in DHM but spore-forming bacteria, such as *B. cereus*, survive the treatment (de Segura et al., 2012; Almutawif et al., 2017). Subsequently, such bacteria can grow in heat-treated milk even faster than in raw milk because of the heat damage to the milk bacteriostatic systems and the absence of competitors (Ford et al., 1977). In agreement with our results, *B. cereus* was the predominant and, often, the only contaminant in those human milk samples that were positive for bacterial growth after Holder pasteurization. *B. cereus* is ubiquitous in nature; it is highly abundant in soil, and it is also adapted to human hosts, either as a pathogen or, more frequently, as a part of the intestinal microbiota of a healthy host (Berthold-Pluta et al., 2015). Additionally, it has been found in milk of healthy rhesus monkeys (Jin et al., 2011), in cows' milk (Magnusson et al., 2007), and in pooled human milk after poor hygienic practices during its manipulation (Decousser et al., 2013). Although any milk bottle with a culture-positive result after pasteurization is rejected by most HMBs, a previous study revealed that all *B. cereus* strains isolated from pasteurized DHM did not possess a high virulence potential (de Segura et al., 2012). However, as the level of virulence is highly variable among different strains (Decousser et al., 2013), caution is strongly required when dealing with this species. Refrigeration or freezing of pasteurized DHM should be strictly applied to control the growth of any remaining microorganism. Although a relative high number of *B. cereus* cells has been usually found in foodborne outbreaks, lower stomach acidity in preterms may allow a higher survival of cells (Stenfors Arnesen et al., 2008; Lotte et al., 2017).

Despite *E. faecalis* is not a spore-forming species, it was isolated from one production batch after HTST processing at 72°C for 5 s but it was completely destroyed after milk processing at 72°C for longer holding times (>5 s) or at 75°C (independently of the holding time). Therefore, the later treatments were efficient in killing any vegetative cell initially present in raw human milk samples. Enterococci, and particularly *E. faecalis*, are natural members of the human and animal intestinal microbiota and are also widespread in the environment. During and shortly after birth, newborn infants are colonized with enterococci (Moles et al., 2015; Gómez et al., 2016) and, although the risk for colonization with multidrug-resistant enterococci increases in preterm newborns, most of them remain uninfected (Hufnagel et al., 2007). However, *E. faecalis* and *Enterococcus faecium* are nosocomial, opportunistic pathogens, known to cause late-onset sepsis in preterm neonates and are notorious for their association with resistance to commonly used antibiotics, such as penicillin, ampicillin, aminoglycosides, and vancomycin (Kumar et al., 2015; Lister et al., 2015). Therefore, raw DHM should receive a heat treatment compatible with the complete killing of these enterococcal species.

Molecular analysis of enterococci isolated before and after the heat treatment showed a variety of strains present in raw DHM. Isolates displaying the same RAPD and PFGE profiles were present in two different batches, which suggest that they shared DHM from the same donors. The isolates that survived the heat treatment (70°C for 5 to 25 s and 72°C for 5 s) were always detected in the raw DHM used for the batch, as confirmed by PFGE. The thermotolerance of *E. faecalis* and *E. faecium* seems to be due to the synthesis of a number of heat shock proteins and, among them, DnaK and GroEL are the best characterized at present (Boutibonnes et al., 1993; Rince et al., 2000; Martínez et al., 2003; Silva Laport et al., 2003). The heat resistance (60–62.5°C, 30 min) of log phase cells of *E. faecalis* grown at 37°C is enhanced by exposing cells to a prior heat shock at 45–50°C for 30 min (Boutibonnes et al., 1993). Similarly, Silva Laport et al. (Silva Laport et al., 2003) reported that at least some *E. faecium* cells could remain viable after 2 h of incubation at 70°C. Globally, it means that the conditions for routine pasteurization of DHM must be chosen very carefully after a tough validation process, such as the described in the present study.

Alkaline phosphatase (ALP) is a membrane–bound glycoprotein that is found in most human tissues and is relevant in clinical chemistry as it serves as indicator of physiological functions and certain diseases. The role of milk ALP in health and nutrition of the newborn infant is uncertain (Bjelakovic et al., 2009). However, it has been used traditionally in dairy industry for the evaluation of milk pasteurization processes due to the close relationship between its thermal resistance and the different time-temperatures treatments required for effective pasteurization (Kay and Graham, 1935). The main advantage is that the result would be available in a short time allowing taking preliminary decisions about the processed batch in the HMB. The usefulness of an enzyme indicator depends not only on the amount of the enzyme present in the milk and the sensitivity of the enzymatic assay, but also on the decimal reductions required for the microbial targets. Although the ALP activity in DHM was variable, no activity could be found either after HTST treatment at both 72 and 75°C. In addition, progressive inactivation was observed at 70°C with increasing treatment time. Therefore, ALP does not seem to be a useful indicator for proper heat treatment, as it is completely destroyed at HTST treatments milder than those required for appropriate bacterial destruction (72°C for at least 10 s).

In dairy industry it has been known for long time that ALP reactivates under certain conditions and alternative indigenous enzymes have been proposed. GGTP, another glycoprotein associated to mammalian tissue and found both in the skim milk fraction and in the milk fat globule membrane, has been the most promising alternative (Fox and Kelly, 2006). GGTP has higher thermal resistance than ALP but its activity is lost gradually at 72°C when processing time increases. In contrast, when DHM was processed at 75°C its activity was absent and, therefore, it could be useful as an indicator of DHM pasteurization. In fact, the reduction of ALP and GGTP activities obtained in this work was similar to that measured by Dhar et al. (1996) in DHM. The activities of both enzymes, ALP and GGTP, can be assayed using simple colorimetric methods and, therefore, could be used as quick, simple, and inexpensive tests. However, additional experiments are needed to confirm its inactivation kinetics before its potential can be exploited.

Heat treatment of milk triggers different chemical reactions involving lactose such as the Maillard reaction, after reaction with proteins, and isomerization. The extent of both reactions depends strongly on the processing conditions (Olano et al., 1989). Lactulose, which is mainly formed by isomerization of lactose, is not present naturally in raw DHM and was not detected in any HTST treated DHM samples in this work, however lactulose was quantified in the HoP DHM samples at similar levels to those found in samples of human milk and colostrum pasteurized by Holder method in other studies (18.96 and 22.62 mg/L respectively) (de Segura et al., 2012; Espinosa-Martos et al., 2013). Lactulose was quantified in commercial HTST pasteurized cow's milk and values ranged from 13.0 to 32.1 mg/L (Montilla et al., 2005). This could be explained by higher intensity of the heat treatments applied in the dairy industry. Previously it was reported that this disaccharide, quantified by using an enzymatic-spectrophotometric method, could be used as a process indicator for commercially pasteurized and direct UHT-treated cow milk samples (Marconi et al., 2004).

On the other hand, Maillard reaction is one of the main reactions causing deterioration of proteins during processing and storage of foods. This reaction can promote nutritional changes such as loss of nutritional quality by destruction of essential amino acids, reduction of protein digestibility and amino acid availability. Furosine is used as an indicator of early stages of Maillard reaction and is considered a useful indicator of the damage in processed foods or foods stored for long periods of time (Corzo-Martinez et al., 2012). In contrast to lactulose, furosine was detected in both Holder-pasteurized and HTST-treated DHM and their concentrations were 2 and 1.5 × times of that found in raw DHM. However, these differences, that indicate the high sensibility of the analytical technique, do not have practical relevance because these levels are below by those reported limits for raw and low pasteurized cow milks (4–14 mg/100 g protein) (Sakkas et al., 2014) and for Holder pasteurized colostrum (13.0–28.6 mg/100 g protein) (Espinosa-Martos et al., 2013). Taken together, the absence of lactulose and the low amount of furosine found in HTST-treated DHM strongly indicate that this heat-treatment does not induce significant heat damage in DHM.

## Conclusion

A new HTST equipment for DHM processing has been designed to ensure accurate, simple and flexible operation at a HMB setting where the available amount of DHM may be variable. In addition, the processing time and labor force, and therefore operational costs, should be reduced significantly in comparison with regular Holder pasteurization. Processing of DHM at 72°C for, at least, 10 s in this HTST system allows to achieve the microbiological safety objectives currently established in HMBs while having a low impact regarding the heat damage of the milk.

Immunological, biochemical and nutritional quality of DHM processed by this new HTST equipment is currently been analyzed in order to evaluate its impact on a large number of compounds with biological activity and nutrient content and compare it to HoP DHM. Therefore, this HTST system will provide efficiently high quality safe pasteurized DHM to preterm and sick infants admitted in most neonatal units.

## Author contributions

DE-V participated in the design of the study, acquisition of the samples, carried out the pasteurization processes, performed the microbiological and enzymatic assays, analyzed and interpreted the data, and drafted the manuscript. IE-M participated in the design of the study and in the data analysis. JR designed the study and provided critical revisions of the manuscript for important intellectual content. NC and AM conducted the lactulose and furosine assays and participated in the data analysis. PS performed the design and operation of the new HTST device. CP-A and LF participated in the design of the study, funding acquisition, analysis of the data and provided a critical revision of the manuscript.

### Conflict of interest statement

The new HTST pasteurization system employed for this study was filed to Spanish Patent and Trademark Office with the title Continuous pasteurizer for human milk, Application No P201531186, filing date 11 August 2015 and PCT/ES2016/070594, filing date 5 August 2016. The authors declare that the research was conducted in the absence of any commercial or financial relationships that could be construed as a potential conflict of interest.

## Footnotes

^1^Grade “A” Pasteurized Milk Ordinance. U.S. Department of Health and Human Services. Public Health Service. Food and Drug Administration (2015). Available online at https://www.fda.gov/downloads/food/guidanceregulation/guidancedocumentsregulatoryinformation/milk/ucm513508.pdf

^2^Donor human milk for low–birth–weight infants. World Health Organization (2017). Available online at: http://www.who.int/elena/titles/donormilk_infants/en/

^3^Anon. Requisite scientific parameters for establishing the equivalence of alternative methods of pasteurization. National Advisory Committee on Microbiological Criteria for Foods (2004). Available online at: https://www.fsis.usda.gov/ophs/nacmcf/2004/NACMCF_Pasteurization_082704.pdf

## Abbreviations

DHM: Donor human milk
HMB: Human Milk Bank
HoP: Holder pasteurization
HTST: High-Temperature Short-Time
ALP: Alkaline phosphatase
GGTP: γ-glutamil transpeptidase
NICU: Neonatal Intensive Care Unit
MOM: Mother's own milk
CIP: Cleaning-In-Place
BHI: Brain Heart Infusion
CNA: Columbia Nalidixic Acid
KAA: Kanamycin Aesculin Azide
MCK: MacConckey.
