# Supplementary material for: High-Temperature Short-Time Pasteurization System for Donor Milk in a Human Milk Bank Setting
Source: Front Microbiol. 2018 May 11;9:926. doi: 10.3389/fmicb.2018.00926 (PMC5958646; doi:10.3389/fmicb.2018.00926)
Supplement: Supplementary file 1 [file Table_1.docx]

Supplementary Material

**High-Temperature Short-Time pasteurization system for donor milk in a human milk bank setting**

**Diana Escuder-Vieco*, Irene Espinosa-Martos, Juan M. Rodríguez, Nieves Corzo, Antonia Montilla, Pablo Siegfried, Carmen Rosa Pallás-Alonso and Leónides Fernández**

*** Correspondence:** Diana Escuder Vieco: [diana.e.vieco@gmail.com](mailto:diana.e.vieco@gmail.com) and Leónides Fernández: [leonides@vetTables.ucm.es](mailto:leonides@vetTables.ucm.es)

## Supplementary Tables

**Supplementary Table 1. Adjustment of DHM flow for each specified treatment time (at the holding section) and calculated lag time for heat-treated DHM sampling after a steady condition for each specified treatment time had been reached.**

| **Fluid flow (L/h)** | **Holding time (s)** | **Lag time for sampling (s)^a^** |
| --- | --- | --- |
| 5 | 25 | 168.14 |
| 7 | 20 | 136.80 |
| 10 | 15 | 95.75 |
| 14 | 10 | 68.40 |
| 28 | 5 | 33.59 |

^a^Lag time was calculated as the time it takes for the DHM to travel from the exit of the holding section to the sampling point at the exit of the system [Lag time = conduction length × conduction section / flow rate; conduction length = 3.83 m; conduction section = 6.9 × 10^-5^ m^2^].

# Supplementary Table 2. Microbial species isolated from raw DHM batches.

|  | **L1** | **L2** | **L3** | **L4** | **L5** | **L6** | **L7** | **L8** | **L9** | **L10** | **L11** | **L12** | **L13** | **L14** |
| --- | --- | --- | --- | --- | --- | --- | --- | --- | --- | --- | --- | --- | --- | --- |
| **Gram-positive bacteria** |  |  |  |  |  |  |  |  |  |  |  |  |  |  |
| *Bacillus* |  |  |  |  |  |  |  | B |  |  |  |  |  |  |
| *Clostridium ramosum* | B M |  |  |  |  |  |  |  |  |  |  |  |  |  |
| *Corynebacterium tuberculostearicum* |  |  |  |  |  |  |  |  |  |  |  | C |  |  |
| *Enterococcus faecalis* | C K | B C K | B C K | B C K | C K | B C K | B C K | B C K | B C K | C K | C K | B C K | B C K | B C K |
| *Staphylococcus aureus* |  |  |  | C | C | B C | B C |  |  | C | C |  |  | C |
| *Staphylococcus epidermidis* | C K | CK | B C K | C K | B C | B C K | B C K | B C K | B C | B C K | B C K | B C K | B C | C |
| *Staphylococcus lugdunensis* |  | B C |  | B | B K |  |  | C K |  |  |  |  | C | B |
| **Gram-negative bacteria** |  |  |  |  |  |  |  |  |  |  |  |  |  |  |
| *Acinetobacter baumannii* complex |  |  |  |  |  |  |  |  | B |  | B | B M |  |  |
| *Acinetobacter ursingii* |  | B |  |  |  | M |  |  |  | B M | M |  | M |  |
| *Chryseobacterium indologenes* | C |  |  |  |  |  |  | C |  |  |  |  |  |  |
| *Enterobacter absuriae/cloacae* |  |  | B M |  |  |  | M | M |  | B M | M |  |  |  |
| *Enterobacter gergoviae* |  |  |  |  |  |  |  |  |  |  | M |  |  |  |
| *Klebsiella sp.* |  |  |  |  |  |  |  | B |  |  |  |  |  |  |
| *Klebsiella oxytoca* |  |  |  |  | M | M |  |  | M |  | B | B M |  | B M |
| *Klebsiella pneumoniae* |  | B M |  |  | M |  | B M |  |  |  |  |  | B M |  |
| *Pantoea agglomerans* |  |  |  |  |  | M |  | M | B K |  |  |  |  |  |
| *Pseudomonas aeruginosa* |  |  |  | B M |  |  |  |  |  |  |  |  |  | B K M |
| *Pseudomonas fluorescens* |  |  |  |  | B M |  | B |  |  |  | B |  |  |  |
| *Pseudomonas putida* |  |  |  |  |  |  |  | B M |  |  |  | B |  |  |
| *Serratia grimessi* |  |  |  |  | B |  |  |  |  |  |  |  |  |  |
| *Serratia liquefaciens* |  |  |  |  |  | B |  |  |  |  |  |  |  |  |
| *Serratia marcenscens* |  | M |  |  |  |  | M |  |  |  |  |  |  |  |
| *Stenotrophomonas maltophilia* | B M |  |  |  |  |  |  |  | M | B |  |  |  | M |
| **Yeasts** |  |  |  |  |  |  |  |  |  |  |  |  |  |  |
| *Candida parapsilosis* |  | C |  |  |  |  |  |  |  |  | C |  |  | C K |
| *Trichosporon asahii* |  |  |  |  |  |  |  |  |  |  |  | C |  |  |
| **Number of different species** | **5** | **7** | **3** | **4** | **8** | **7** | **7** | **9** | **6** | **6** | **10** | **7** | **5** | **8** |

List of microbial species isolated from 14 raw DHM batches used for HTST processing at 70ºC (L1 to L5), 72ºC (L6 to L10) and 75ºC (L11 to L14). The letter inside the dark square indicates the culture media where the microorganism was isolated (B, Brain Hearth Infusion; C, Columbia Nalidixic Acid; K, Kanamycin Aesculin Azide, and M, MacConkey agar plates). At the bottom the number of different microbial species isolated form each raw DHM processing batch is indicated.

**Supplementary Table 3.** **Microbiological analysis of the 14 DHM production batches before and after HTST and HoP treatments.**

| **Heat treatment** |  | **Total counts BHI^a^** |  | **Total counts CNA^a^** |  | **Total counts KAA^a^** |  | **Total counts MCK^a^** |
| --- | --- | --- | --- | --- | --- | --- | --- | --- |
|  | **n/N^b^** | **(mean ± SD)^c^** | **n/N** | **(mean ± SD)** | **n/N** | **(mean ± SD)** | **n/N** | **(mean ± SD)** |
| Raw | 5/5 | 4.98 ± 0.94 | 5/5 | 4.31 ± 0.73 | 5/5 | 4.06 ± 0.95 | 5/5 | 4.11 ± 1.83 |
| HTST 70ºC, 5 s | 2/5 | 2.60 ± 0.30 | 3/5 | 2.24 ± 0.50 | 1/5 | 1.70 | 0/5 | ‒ |
| HTST 70ºC, 10 s | 2/5 | 2.62 ± 0.60 | 2/5 | 2.06 ± 0.83 | 1/5 | 1.47 | 0/5 | ‒ |
| HTST 70ºC, 15 s | 2/5 | 2.27 ± 0.03 | 2/5 | 1.83 ± 0.75 | 1/5 | 1.30 | 0/5 | ‒ |
| HTST 70ºC, 20 s | 2/5 | 2.04 ± 0.13 | 2/5 | 1.88 ± 0.40 | 0/5 | ‒ | 0/5 | ‒ |
| HTST 70ºC, 25 s | 2/5 | 2.09 ± 0.07 | 1/5 | 2.30 | 0/5 | ‒ | 0/5 | ‒ |
| Holder (62.5ºC, 30 min) | 1/5 | 2.07 | 0/5 | ‒^d^ | 0/5 | ‒ | 0/5 | ‒ |
| Raw | 5/5 | 4.92 ± 0.40 | 5/5 | 4.67 ± 0.40 | 5/5 | 4.08 ± 0.73 | 5/5 | 4.17 ± 0.89 |
| HTST 72ºC, 5 s | 2/5 | 2.27 ± 0.03 | 0/5 | ‒ | 1/5 | 1.30 | 0/5 | ‒ |
| HTST 72ºC, 10 s | 2/5 | 2.40 ± 0.14 | 0/5 | ‒ | 0/5 | ‒ | 0/5 | ‒ |
| HTST 72ºC, 15 s | 2/5 | 2.43 ± 0.12 | 0/5 | ‒ | 0/5 | ‒ | 0/5 | ‒ |
| HTST 72ºC, 20 s | 2/5 | 2.55 ± 0.03 | 0/5 | ‒ | 0/5 | ‒ | 0/5 | ‒ |
| HTST 72ºC, 25 s | 2/5 | 2.57 ± 0.00 | 0/5 | ‒ | 0/5 | ‒ | 0/5 | ‒ |
| Holder (62.5ºC, 30 min) | 2/5 | 2.36 ± 0.02 | 0/5 | ‒ | 0/5 | ‒ | 0/5 | ‒ |
| Raw | 4/4 | 4.28 ± 0.70 | 4/4 | 4.17 ± 0.62 | 4/4 | 3.73 ± 1.17 | 4/4 | 3.50 ± 1.04 |
| HTST 75ºC, 5 s | 1/4 | 2.23 | 0/4 | ‒ | 0/4 | ‒ | 0/4 | ‒ |
| HTST 75ºC, 10 s | 1/4 | 2.20 | 0/4 | ‒ | 0/4 | ‒ | 0/4 | ‒ |
| HTST 75ºC, 15 s | 1/4 | 1.95 | 0/4 | ‒ | 0/4 | ‒ | 0/4 | ‒ |
| HTST 75ºC, 20 s | 1/4 | 2.07 | 0/4 | ‒ | 0/4 | ‒ | 0/4 | ‒ |
| HTST 75ºC, 25 s | 1/4 | 2.17 | 0/4 | ‒ | 0/4 | ‒ | 0/4 | ‒ |
| Holder (62.5ºC, 30 min) | 1/4 | 2.23 | 1/4 | 1.30 | 0/4 | ‒ | 0/4 | ‒ |

^a^Growth media where the isolates were recovered: BHI (Brain Hearth Infusion), CNA (Columbia Nalidixic Agar), KAA (Kanamycin Aesculin Agar).

^b^n/N, number of DHM batches where microbial counts were enumerated by plate counting (n) out of the total batches processed at that temperature (N).

^c^Total microbiological counts (log_10_ cfu/mL).

^d^Below the detection limit of the method (˂1.30 log_10_ cfu/mL)
